# TRIM22 inhibits the proliferation of gastric cancer cells through the Smad2 protein

**DOI:** 10.1038/s41420-021-00627-5

**Published:** 2021-09-07

**Authors:** Zhuqing Zhou, Wei Gao, Biao Yuan, Shun Zhang, Kaijing Wang, Tao Du

**Affiliations:** 1 Research Center for Translational Medicine at East Hospital, School of Life Science and Technology, Tongji University, Shanghai, 200092 China; 2 Department of Gastrointestinal Surgery, Shanghai East Hospital, School of Medicine, Tongji University, Shanghai, 200092 China

**Keywords:** Gastrointestinal cancer, Tumour-suppressor proteins

## Abstract

TRIM22 is involved in tumorigenesis and development, but its mechanism is not clear. In this study, we investigated the expression and biological role of TRIM22 in gastric cancer. We found that TRIM22 mRNA and protein expression was abnormally low in gastric cancer tissues and cells and correlated with tumor size and depth of invasion. Overexpression of TRIM22 significantly inhibited the proliferation, colony formation, and migration of gastric cancer cells and downregulated the expression of HSPA6. However, the HSPA6-siRNA complementation test showed that TRIM22 did not regulate cell proliferation through HSPA6. Furthermore, overexpression of TRIM22 downregulated the phosphorylation of Smad2 and Smad3. In addition, TRIM22 directly binds to Smad2, and overexpression of Smad2 can reverse the inhibition of cell proliferation and migration induced by TRIM22. In vivo, overexpression of TRIM22 significantly inhibited the growth of subcutaneous xenografts in nude mice. Our study indicates that TRIM22 has an important role in the development of gastric cancer and may inhibit the proliferation of gastric cancer cells through Smad2.

## Background

Gastric cancer is one of the most common malignant tumors worldwide and has a poor prognosis. The 5-year survival rate of gastric cancer is only 45%, and most patients experience recurrence within 2 years [1, 2]. The occurrence and development of gastric cancer involve multiple genes with complex molecular mechanisms. Therefore, it is of great clinical significance to explore the molecular mechanisms related to gastric cancer cell proliferation.

Tripartite motif-containing 22 (TRIM22) is a member of the tripartite motif (TRIM) family of proteins and is interferon (IFN)-inducible [3]. IFN-γ can significantly stimulate the expression of TRIM22, which is also a target gene of p53 [4]. TRIM22 was reported to activate E3 ubiquitination ligase, and could also ubiquitinate itself in vitro [5]. TRIM22 is mostly located in the cytoplasm but can be transported to the nucleus in some cases [6, 7]. TRIM22 is an important regulator of the innate immune response against pathogens, mainly acting as an inhibitor of the replication of a variety of viruses, including Encephalomyocarditis virus (EMCV), HBV, and HIV-1 [8–10]. It is well established that TRIM22 can inhibit HBV replication by interfering with HBV core promoter [11], and inhibit HIV replication by preventing transcription factor specific protein 1 (SP1) from binding to HIV promoter [12]. Recent studies have shown that TRIM22 is also involved in cell differentiation and proliferation and might play a role in the development of some tumors. TRIM22 expression was upregulated in non-small-cell lung cancer and promoted the proliferation, colony formation, and invasion of A549 cells while inducing epithelial–mesenchymal transition (EMT) via the Akt/GSK-3β/β-catenin signaling pathway [13]. Knockdown of TRIM22 expression inhibited proliferation and invasion and significantly induced cell cycle arrest by regulating CDK4, cyclin D1, P70S6K, and p53 expression in chronic myelogenous leukemia cells [14]. These results suggest that TRIM22 may have a role in tumorigenesis and development. However, the role of TRIM22 in gastric cancer remains unclear.

Uncontrolled proliferation of gastric cancer cells is a process involving multigene and multistep regulation. Previous studies have shown that the Smad-mediated TGF-β pathway has an important role in tumorigenesis, participates in the regulation of cell cycle progression and apoptosis, and can promote cell invasion and EMT in tumor cells [15–17]. For example, TGF-β activated the expression of cyclin-dependent kinase inhibitors p15 (Ink4B) and p21 (WAF1/Cip1) and repressed the expression of c-Myc, thus inhibiting cell proliferation [18]. In this study, we measured the expression of TRIM22 in gastric cancer tissues and cells and investigated its effect on cell proliferation using a lentiviral-mediated overexpression approach. Finally, we explored the molecular mechanism of TRIM22-mediated regulation of cell proliferation.

## Results

### TRIM22 expression is downregulated in gastric cancer tissues and cells

**Fig. 1:**
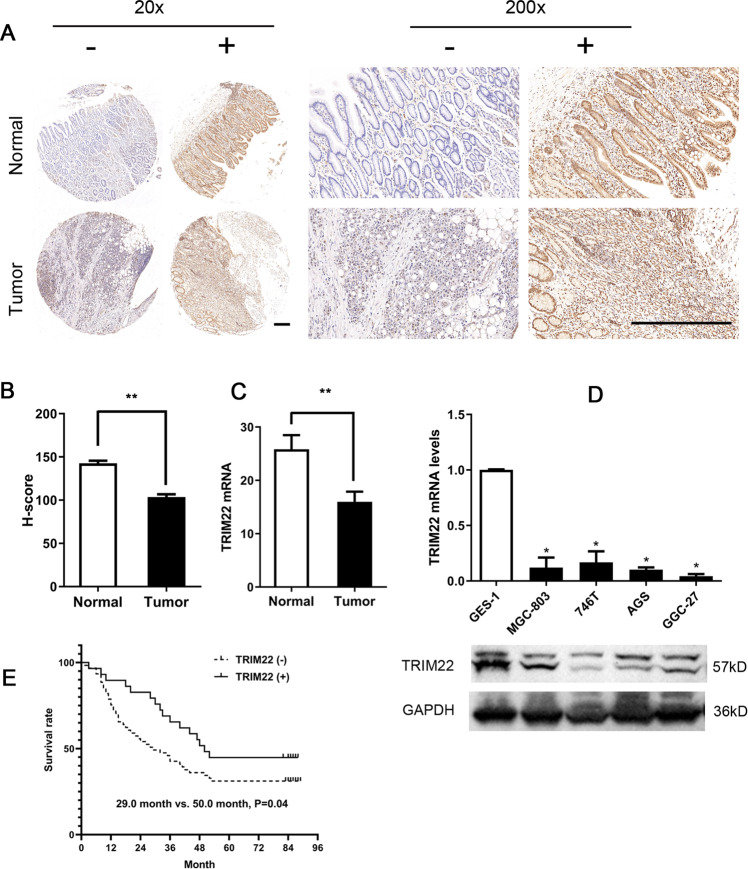
TRIM22 expression is downregulated in gastric cancer tissues and cells. **A** Immunohistochemical staining image of TRIM22 in gastric cancer and adjacent tissues (magnification: ×20 and ×200, scale bars = 200 μm). **B** H-score of TRIM22 in gastric cancer and adjacent tissues (*n* = 90, **P < 0.01). **C** Expression of TRIM22 mRNA (qPCR, **P < 0.01). **D** Expression of TRIM22 mRNA and protein in gastric cancer cell lines (qPCR and WB, *P < 0.01). **E** Kaplan–Meier survival curve analysis of TRIM22 in gastric cancer. Data are shown as mean ± SD of three independent experiments.

The expression of TRIM22 protein in 90 pairs of gastric cancer and adjacent tissues was measured by immunohistochemistry. TRIM22 protein was localized in the cytoplasm and nucleus (Fig. 1A). TRIM22 protein expression was downregulated in gastric cancer tissue compared to the adjacent group, and the H-score was 103.6 ± 30.5 vs 142.6 ± 28.8, with a significant difference (*P* < 0.01, Fig. 1B). TRIM22 mRNA expression was also significantly downregulated in gastric cancer tissues (*P* < 0.05, Fig. 1C). Furthermore, we divided gastric cancer tissues into a TRIM22-positive group (*n* = 27) and a TRIM22-negative group (*n* = 63). TRIM22 protein expression was related to tumor size and depth of invasion (*P* < 0.05, Table 1). We also found that the expression of TRIM22 protein and mRNA decreased in gastric cancer cell lines compared with that in the immortalized normal gastric epithelial cell line GES-1 (*P* < 0.01, Fig. 1C).

**Table 1:** Relationship between TRIM22 expression level and clinicopathologic variables in 90 gastric cancer tissues.

Clinicopathologic parameters	TRIM22 protein		*P*
	−	+	
	(*n* = 63)	(*n* = 27)	
*Age (years)*			
≤60	25	9	0.569
>60	38	18	
*Gender*			
Male	36	20	0.129
Female	27	7	
*Tumor size (cm)*			
≤5	40	23	0.04
>5	23	4	
*Lauren classification*			
Intestinal	27	15	0.268
Diffuse	36	12	
*Differentiation*			
Well, moderately	25	11	0.925
Poorly, undifferentiated	38	16	
*Local invasion*			
T1, T2	14	19	<0.01
T3, T4	49	8	
*Lymph node metastasis*			
No	11	5	0.904
Yes	52	22	
*TNM stage*			
I, II	24	14	0.226
III, IV	39	13	

In addition, Kaplan–Meier survival analysis showed that patients who had TRIM22-positive gastric cancer had a better prognosis than those with TRIM22-negative cancer with a median survival of 50.0 months vs. 29.0 months. (*P* < 0.05, Fig. 1D).

### Overexpression of TRIM22 inhibits the proliferation and migration of gastric cancer cells in vitro

**Fig. 2:**
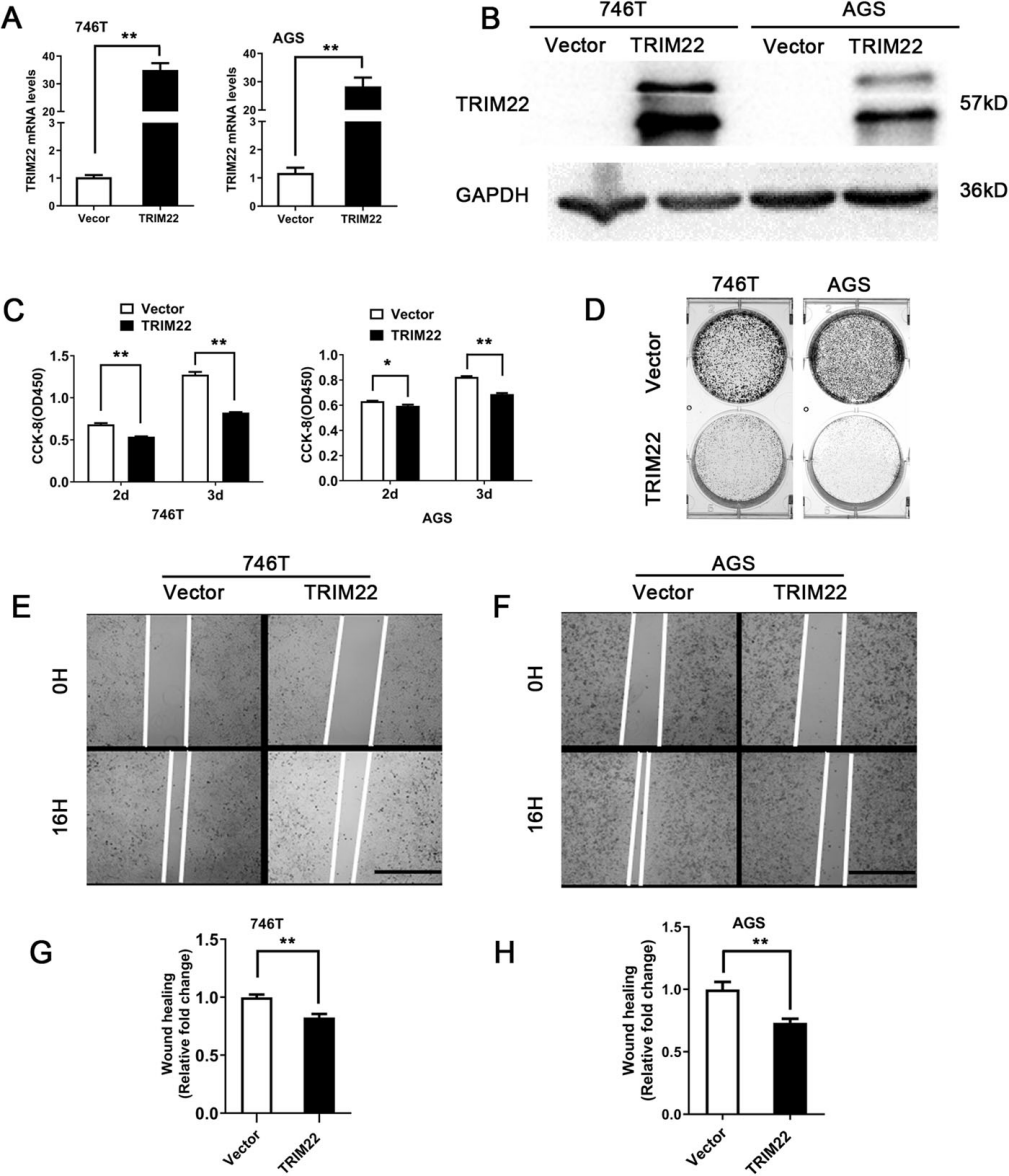
TRIM22 overexpression inhibited the proliferation and migration of gastric cancer cells in vitro. **A**, **B** Validation of the transfection efficiency of TRIM22 (qPCR and WB, *P < 0.01). 746 T and AGS cells were transfected with TRIM22 lentivirus to construct stably transfected cells. **C**, **A** CCK-8 assay was used to detect cell proliferation (*P < 0.01). **D** colony formation assay. **E**, **F** A wound-healing assay was used to detect wound-healing ability. **G**, **H** Statistics of wound-healing area (**P < 0.01, scale bars = 1000 μm). Data are shown as mean ± SD of three independent experiments.

To further study the biological function of TRIM22 in cells, we constructed TRIM22-overexpressing cells using lentiviral transfection. The qPCR and WB results showed that the expression of TRIM22 was significantly increased in 746T-TRIM22 and AGS-TRIM22 cells compared to the control (*P* < 0.01, Fig. 2A, B). The CCK-8 assay showed that TRIM22 overexpression significantly inhibited the proliferation of 746 T and AGS cells compared with the controls (*P* < 0.05, Fig. 2C). Colony formation assays revealed that the number of clones capable of forming individual colonies in the TRIM22 overexpression group was significantly decreased (Fig. 2D). In addition, wound-healing assays showed that the overexpression of TRIM22 significantly inhibited the wound-healing capabilities of 746 T and AGS cells (*P* < 0.01, Fig. 2E–H).

### Analysis of downstream genes possibly regulated by TRIM22

**Fig. 3:**
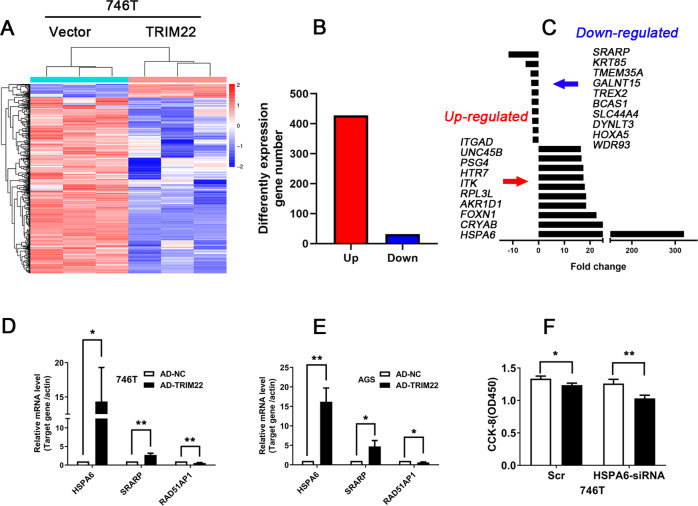
The effect of TRIM22 overexpression on gene expression in gastric cancer cells. **A** Thermogram of differentially expressed genes caused by TRIM22 overexpression. The analysis was performed by RNA-Seq. **B**, **C** Statistical analysis of differential gene mRNA expression. **D**, **E** Verification of mRNA expression in 746 T and AGS cells (qPCR, **P < 0.01). **F** Effect of co-transfection of HSPA6-siRNA on proliferation in 746T-TRIM22 cells (CCK-8 assay, *P < 0.05, **P < 0.01). Data are shown as mean ± SD of three independent experiments.

To further identify the downstream genes of TRIM22 in gastric cancer, we analyzed the differentially expressed genes between TRIM22-overexpressing and control cells using RNA-Seq. The results showed that the expression of 428 genes was significantly increased and the expression of 32 genes was significantly decreased in TRIM22-overexpressing cells (Fig. 3A, B). Among these genes, the expression of HSPA6 and SRARP showed the most significant difference, with a 320-fold increase and a 0.09-fold decrease, respectively (Fig. 3C). Subsequently, we further detected the expression of these genes in 746T-and AGS cells overexpressing TRIM22 using qPCR. The results showed that HSPA6, SRARP, and RAD51AP1 were differentially expressed (Fig. 3D, E, *P* < 0.01). However, a CCK-8 assay showed that HSPA6-siRNA could not rescue the inhibition of cell proliferation induced by TRIM22 overexpression in 746 T cells (Fig. 3F). Similarly, SPARP-siRNA and RAD51AP1-overexpression plasmids could not reverse the inhibitory effect of TRIM22 (data not shown). These results suggested that TRIM22 might not regulate cell proliferation via HSPA6, SRARP, and RAD51AP1.

### TRIM22 can inhibit the phosphorylation of Smad Protein

**Fig. 4:**
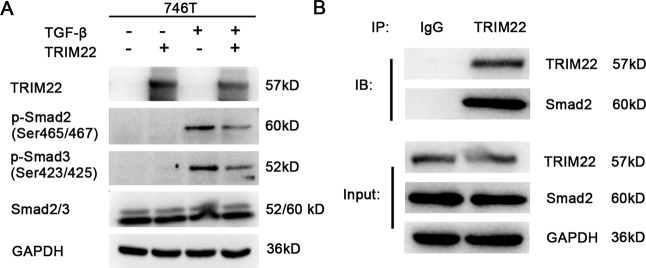
Effect of TRIM22 on Smad protein expression. **A** Overexpression of TRIM22 inhibited TGF-β-induced phosphorylation of Smad2 and Smad3 (WB). **B** TRIM22 could direct binding to Smad2 (Co-IP). Data are shown as mean ± SD of three independent experiments.

Next, we explored changes in genes downstream of TRIM22, resulting from TRIM22 overexpression using WB. We found that TRIM22 overexpression inhibited the TGF-β-induced phosphorylation of Smad2 and Smad3 (Fig. 4A). Immunoprecipitation showed direct binding between TRIM22 and Smad2 (Fig. 4B).

### Overexpression of Smad2 can rescue the inhibitory effect of TRIM22 on cell proliferation and migration

**Fig. 5:**
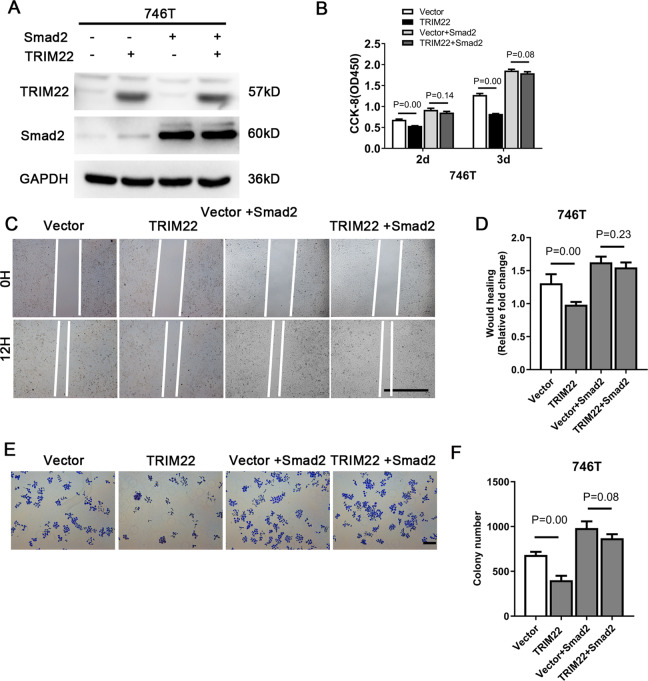
Smad2 overexpression rescued the proliferation and migration effects of TRIM22 overexpression. **A** Protein expression in TRIM22 and Smad2 cotransfected cells (WB). **B** CCK-8 assay. **C**, **D** Wound healing assay, scale bars = 1000 μm. **E**, **F** Colony formation assay, scale bars = 100 μm. Data are shown as mean ± SD of three independent experiments.

Because TRIM22 could inhibit Smad2 phosphorylation, we speculated that TRIM22 might regulate the proliferation and migration of gastric cancer cells through Smad2. Therefore, we used a Smad2 plasmid to transfect 746T-TRIM22 cells and found that Smad2 protein expression increased significantly (Fig. 5A). Next, CCK-8, wound-healing and colony formation assays all showed that overexpression of Smad2 could rescue the proliferation and migration effect of TRIM22. These results suggest that TRIM22 may regulate the proliferation and migration of gastric cancer cells by inhibiting the phosphorylation of Smad2.

### TRIM22 overexpression inhibits tumor proliferation in vivo

**Fig. 6:**
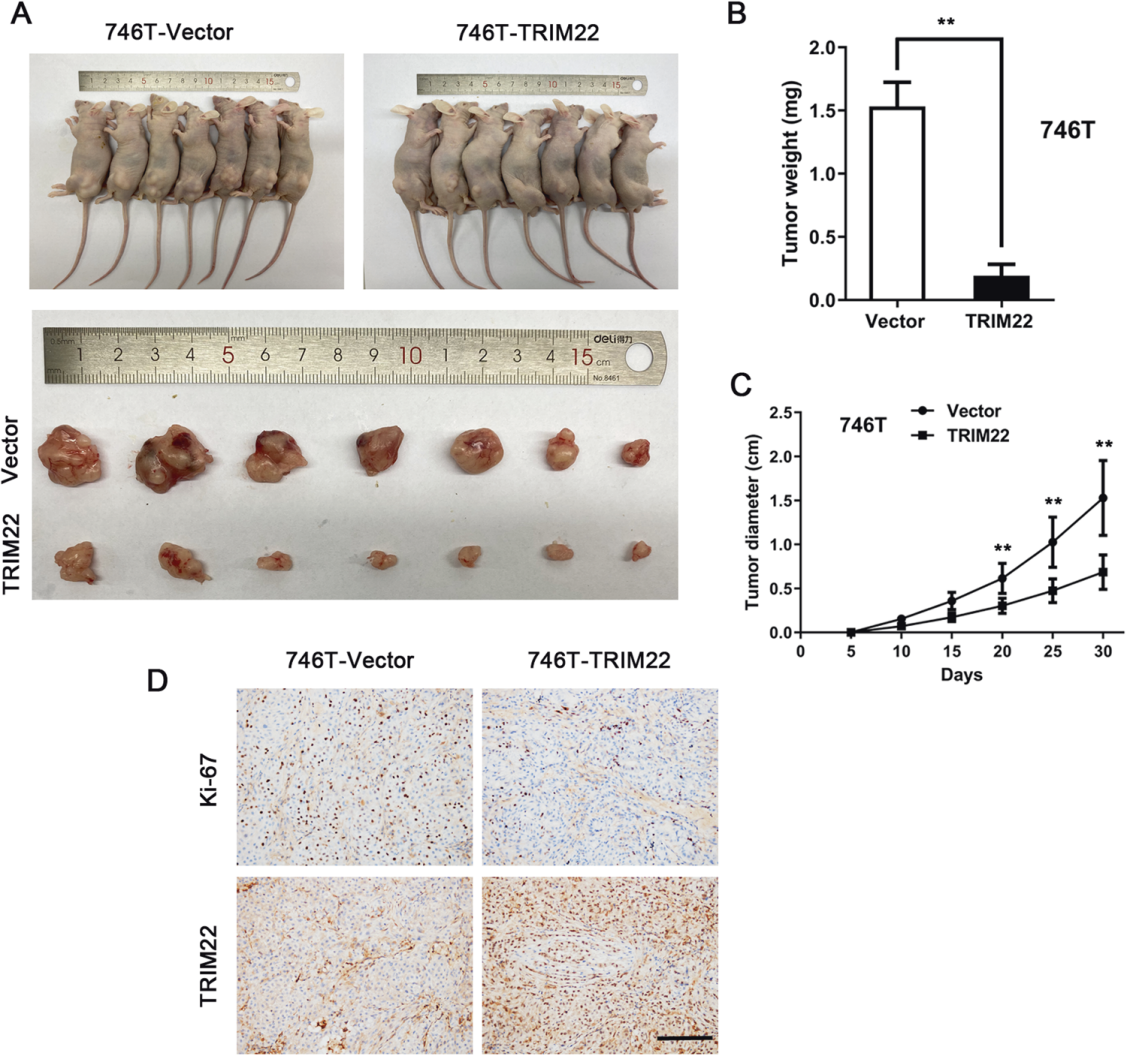
TRIM22 inhibited the proliferation of gastric cancer cells in vivo. **A** Image of subcutaneously transplanted tumors in nude mice. **B** Analysis of tumor weight (**P < 0.01). **C** Tumor growth curve. **D** Immunohistochemical staining image of Ki-67 protein, Scale bars = 100 μm. Data are shown as mean ± SD of three independent experiments.

To investigate the effect of TRIM22 in vivo, 746 T cells stably expressing TRIM22 or control cells were injected into nude mice to establish a subcutaneous xenograft tumor model. The mice were skilled 30 days later. We found that the growth of subcutaneous xenografts in mice injected with cells overexpressing TRIM22 was significantly inhibited in comparison with the growth of the control cell-derived xenografts (Fig. 6A–C, *P* < 0.01). Immunohistochemical staining showed that Ki-67 protein expression was significantly downregulated in the TRIM22-overexpressing tumors (Fig. 6D).

## Discussion

The main cause of tumorigenesis is the inactivation of oncogenes and the loss of tumor suppressor genes, which leads to a series of downstream signal changes and uncontrolled cell proliferation. Earlier studies have shown that the main function of TRIM22 is to recognize viruses, inhibit viral replication, activate immune cells, and regulate ubiquitination in vivo. Recent studies have reported that TRIM22 plays a role in lung cancer and leukemia, which indicates that TRIM22 may be involved in the regulation of malignant tumors. The biological function and specific mechanism of TRIM22 in cancer have not been clearly described in the existing literature. Therefore, this study aimed to explore whether TRIM22 is involved in the regulation of the occurrence and development of gastric cancer.

The results of qPCR and immunohistochemistry showed that the expression of TRIM22 mRNA and protein was abnormally decreased in gastric cancer, as well as in gastric cancer cells, which indicated that there was an abnormal transcription of the TRIM22 gene in gastric cancer. The expression of TRIM22 was related to tumor size and depth of invasion in tumor tissues. Further cellular functional assays showed that TRIM22 overexpression significantly inhibited the proliferation and migration of gastric cancer cells, which suggested that TRIM22 may be a tumor suppressor gene and participate in the regulation of the occurrence and development of gastric cancer. However, it should be noted that previous studies have identified TRIM22 as an oncogene in lung cancer and leukemia, which suggests that the transcriptional regulation mechanism of TRIM22 is rather complex.

To further explore the molecular mechanism by which TRIM22 inhibits cell proliferation, we analyzed changes in genes downstream of TRIM22 resulting from TRIM22 overexpression using RNA-Seq. We screened several differentially expressed genes, among which HSPA6 dominated. HSPA6, also known as heat shock protein 70 member 6 (hsp70b’), is a member of the heat shock protein family [19], which plays an important role in maintaining cell tolerance to injury and stress [20, 21]. Recent studies have found that HSPA6 can enhance the inhibitory effect of garlic extract on the proliferation, migration, and invasion of bladder cancer cells by enhancing the ATM-CHK2-Cdc25C-p21 WAF1-Cdc2 cascade response and MAPK and Akt phosphorylation and inhibiting MMP-9 expression [22]. Subsequently, we performed qPCR to verify the expression of these genes in gastric cancer cells. The results showed that HSPA6 and SPARP expression was significantly upregulated in TRIM22-overexpressing cells, while RAD51AP1 expression was downregulated. These results suggest that TRIM22 may be involved in the regulation of HSPA6, SPARP, and RAD51AP1 expression. However, further CCK-8 assays showed that HSPA6 knockdown did not reverse the proliferation effect induced by TRIM22 overexpression, which was similar to SPARP and RAD51AP1. These results suggest that TRIM22 may not have a biological function through HSPA6, SPARP, and RAD51AP1 but instead through other mechanisms. Our results may reveal new downstream target genes of TRIM22 and we speculate that TRIM22 may play an important role in cell stress, which is also a future research direction.

As protein phosphorylation is one of the main mechanisms of gene regulation, we analyzed changes in protein phosphorylation caused by TRIM22 overexpression. The results showed that overexpression of TRIM22 could inhibit the TGF-β-induced phosphorylation of Smad2 and Smad3. Immunoprecipitation assays showed that there was a direct interaction between TRIM22 and Smad2. Furthermore, overexpression of Smad2 reversed the proliferation and migration effects of TRIM22. As mentioned above, the TGF-β/Smad signaling pathway is involved in important cellular processes in vivo, including cell proliferation, differentiation, metastasis, cell survival, and epithelial–mesenchymal transition (EMT) [15–17]. Studies have shown that the activation of TG-β/Smad signaling has dual effects in different stages of tumors and has an antitumor role by inducing cell cycle arrest and apoptosis in the early stage of tumor development [23–25]. However, in the advanced stage, the mutation or deletion of signaling molecules could lead to the loss of tumor inhibitory activity [26]. For example, Smad2 and Smad3 mutations have been found in colon cancer, pancreatic cancer, and other tumors [27–29]. Phosphorylated Smad2 and Smad3 can bind to Smad4, and this complex can enter the nucleus, which is critical for TGF-β/Smad signal transduction [30]. As TRIM22 can enter the nucleus and bind to transcription factors or downstream proteins in a specific environment, it is reasonable to speculate that TRIM22 may affect Smad2/3 directly or indirectly, thereby regulating downstream signaling. There are also some shortcomings in this study as we did not further clarify the interaction of TRIM22 and Smad2, the changes in downstream molecules of the TGF-β/Smad pathway, and whether it regulates the progression of EMT.

In conclusion, our results showed that TRIM22 expression was abnormally downregulated in gastric cancer and that TRIM22 could inhibit the proliferation and migration of gastric cancer cells by affecting Smad2 protein phosphorylation. Our results extend the understanding of TRIM22 functions, especially in the field of cancer.

## Materials and methods

### Ethical statement

This study was approved by the ethics committee of Shanghai East Hospital (Permit number: 2020-154). All animal experiments were approved by the Experimental Animal Ethics Committee of Shanghai East Hospital and performed according to the Guide for the Institutional Animal Care and Use Committee of Shanghai Tongji University (Shanghai China).

### Tissues

The specimens were collected from patients who underwent gastric cancer surgery in Shanghai East hospital affiliated with Tongji University from January 2012 to December 2014. Written informed consent was obtained from all participants. All patients did not receive chemotherapy or radiotherapy before operation and were confirmed as gastric cancer by postoperative pathology. International TNM staging was used.

### Cells and chemicals

Gastric cancer cell lines MGC803, 746 T, AGS, GGC-27, and immortalized normal gastric mucosa cell line GES-1 were preserved in the laboratory. RPMI-1640 medium (GIBCO BRL, Gaithersburg, MD, USA) containing 100 μ/ml penicillin, 100 μg/ml streptomycin, and 10% fetal bovine serum were used in the culture at 37 °C in the air with 5% CO_2_ saturation. TGF-β was purchased from R&D system.

### Plasmids construction and transfection

TRIM22 lentivirus was from Shanghai Genechem. Smad2 plasmid was from Santa Cruz (Santa Cruz, CA, USA). The target sequence of HSPA6- siRNA was 5′-CCACUUCAUGGAAGAAUUCTT-3′, and the negative control sequence was 5′-UUCUCCGAACGUGUCACGUTT-3′, and siRNA was transfected using Lipofectamine RNAiMAX (Invitrogen, CA, USA).

### Immunohistochemical staining

Gastric cancer tissue microarray was detected using the highly sensitive streptavidin-biotin-peroxidase immunohistochemical method. Anti TRIM22 polyclonal antibody (Abcam, MA, USA) was diluted with 1:100. The images were scanned by pannoramic MIDI (3DHISTECH, Budapest, Hungary). H-score was performed by Quant center software [31]. The intensity of staining was divided into four grades: strong staining, moderate staining, weak staining, and no staining. H-score = ∑(PI × I) = (weak staining percentage) × 1 + (medium positive staining) × 2 + (strong positive staining) × 3.

The positive expression of TRIM22 was judged by the presence of brown granules in the cytoplasm and nucleus. The percentage of positive cells was divided into five grades (score): <5% (0), 5–25% (1), 25–50% (2), 50–75% (3), 75–100% (4). The TRIM22 staining was determined by percentage score × intensity score. The overall score of ≤3 was defined as negative, and positive for >3.

### QRT-PCR

Total RNA was extracted with Trizol (Invitrogen) and cDNA was synthesized with a reverse transcription kit (Promega, WI, USA) according to the instructions. QRT-PCR was performed using SBGREEN PCRmaster mix (ABI, FL, USA) and detected by Applied Biosystems 7500 fast sequence detection system (Life Technologies Corporation, CA, USA). GAPDH was used as an internal reference. The relative expression of genes was evaluated by the value of relative CT (threshold cycle), and the relative expression level of mRNA was calculated by 2^-ΔCT^. The primers were as followed: TRIM22: 5′-CTTTATGGCTGTGCCTCCC-3′ (fwd) and 5′-GTAGATGAGTGCTCCGTGGTT-3′ (rev), HSPA6: 5′-GAGGTGGAGAGGATGGTTCA-3′ (fwd) and 5′-TGTCCTCTTCGGGAATCTTG-3′ (rev), SRARP: 5′-CGTGTTCTGTGGGGAAAACT-3′ (fwd) and 5′- GGGCTTTCAGTGAGTCCTTG-3′ (rev), RAD51AP1: 5′-GACTTCGGTGGACTCTGCTC-3′ (fwd) and 5′-CGGAGACTCTGATTGGGAGA-3′ (rev), GAPDH: 5′-TTGGCATCGTTGAGGGTCT-3′ (fwd) and 5′- CAGTGGGAACACGGAAAGC-3′ (rev).

### Immunoblotting

Cells were lysed by high efficient RIPA plus protease inhibitor (Conway, Shanghai, China) and phosphatase inhibitor (CST, MA, USA). The protein concentration was measured by assay kit (Pierce, IL, USA). Protein was transferred to the polyvinylidene fluoride membrane after electrophoresis and blocked by a TBST buffer containing 5% non-fat dry milk. TRIM22 antibody was from Abcam. Smad2/3, p-Smad2, p-smad3, and IgG antibody were from CST, Ki-67 antibody was from Dako (Copenhagen, Denmark), GAPDH antibody was from Kangcheng (Shanghai, China). Rabbit and mouse secondary antibodies were from CST.

### CCK-8 cell proliferation experiment

Cells were cultured in a 96-well plate at a concentration of 1 × 10^4^/ml. One hundred microliter CCK-8 (Dojindo, Kumamoto, Japan) was added to the plate every 24 h and OD450 was measured after 0d, 2d, and 3d.

### Wound-healing assay

Cells were cultured in a six-well plate, scratched with a pipette tip, and change medium to Dulbecco’s Modified Eagle Medium with 1% serum (GIBCO). The wound was imaged after 0 h and 16 h.

### Clonal formation assay

Cells were seed into 10 cm dish with 1000 cells and 10 ml medium per well. Cells were then cultured for 10 days until clones were formed. Cell clones were fixed with methanol, stained with crystal violet, and counted under the microscope.

### RNA-Seq

Total RNA was extracted from 746 T cells and control cells overexpressing TRIM22. After quality detection and RNA fragmentation, cDNA was generated by reverse transcription and then amplified by PCR. The constructed library was sequenced by Illumina HiSeq TM 2500 sequencer.

### Co-immunoprecipitation

Cellular protein was collected by using the IP Buffer (Thermo Fisher Scientific, MA, USA). Protein lysates of (200 mg) were immunoprecipitated with primary antibodies or control IgG using Dynabeads Coimmunoprecipitation Kit (Thermo Fisher Scientific) and then subjected to electrophoresis on a sodium dodecyl sulphate-polyacrylamide gel. Equal amounts of protein (30 mg) were loaded on the gel and subjected to WB assay. The target proteins were probed with the primary antibodies followed by corresponding secondary antibodies.

### In vivo tumorigenesis

Male BALB/C nude mice (Institute of Zoology, Chinese Academy of Sciences, Shanghai, China) were housed in an SPF (specific pathogen-free) environment in the experimental animal center of Shanghai East hospital affiliated to Tongji University. The research was carried out according to the experimental animal law and the guidelines of the institute. In all, 1 × 10^6^ cells were injected subcutaneously into 4-week-old male nude mice (seven in each group). The tumor diameter was measured every 7 days. The tumor volume was calculated according to the formula: volume = (*W*^2^ × *L*)/2. Mice were sacrificed under anesthesia after 30 days.

### Statistical analysis

The data were shown as mean ± SD. The expression of TRIM22 and clinical parameters was analyzed by Pearson *χ*^2^ experience test. Student’s *t* test was used to compare the experimental groups. Kaplan–Meier survival analysis was used to compare survival curves. A two-tailed value of *P* < 0.05 was considered statistically significant, and the data were analyzed by SPSS 23.0 software.

## Data Availability

The data used to support the findings of this study are available from the corresponding author upon reasonable request.

## References

[1] Erratum: Global cancer statistics 2018: GLOBOCAN estimates of incidence and mortality worldwide for 36 cancers in 185 countries. CA Cancer J Clin. 2020;70:313. 10.3322/caac.21609 32767693

[2] Yang HK, Berlth F (2019). Gastric cancer surgery: the importance of technique and not only the extent of lymph node dissection. Lancet Oncol.

[3] Kelly JN, Woods MW, Xhiku S, Barr SD (2014). Ancient and recent adaptive evolution in the antiviral TRIM22 gene: identification of a single-nucleotide polymorphism that impacts TRIM22 function. Hum Mutat.

[4] Sawyer SL, Emerman M, Malik HS (2007). Discordant evolution of the adjacent antiretroviral genes TRIM22 and TRIM5 in mammals. PLoS Pathog.

[5] Duan Z, Gao B, Xu W, Xiong S (2008). Identification of TRIM22 as a RING finger E3 ubiquitin ligase. Biochem Biophys Res Commun.

[6] Sivaramakrishnan G, Sun Y, Rajmohan R, Lin VC (2009). B30.2/SPRY domain in tripartite motif-containing 22 is essential for the formation of distinct nuclear bodies. FEBS Lett.

[7] Herr AM, Dressel R, Walter L (2009). Different subcellular localisations of TRIM22 suggest species-specific function. Immunogenetics.

[8] Eldin P, Papon L, Oteiza A, Brocchi E, Lawson TG, Mechti N (2009). TRIM22 E3 ubiquitin ligase activity is required to mediate antiviral activity against encephalomyocarditis virus. J Gen Virol.

[9] Lim KH, Park ES, Kim DH, Cho KC, Kim KP, Park YK (2018). Suppression of interferon-mediated anti-HBV response by single CpG methylation in the 5′-UTR of TRIM22. Gut.

[10] Saito-Kanatani M, Urano T, Hiroi H, Momoeda M, Ito M, Fujii T (2015). Identification of TRIM22 as a progesterone-responsive gene in Ishikawa endometrial cancer cells. J. Steroid Biochem Mol Biol.

[11] Gao B, Duan Z, Xu W, Xiong S (2009). Tripartite motif-containing 22 inhibits the activity of hepatitis B virus core promoter, which is dependent on nuclear-located RING domain. Hepatology.

[12] Turrini F, Marelli S, Kajaste-Rudnitski A, Lusic M, Van Lint C, Das AT (2015). HIV-1 transcriptional silencing caused by TRIM22 inhibition of Sp1 binding to the viral promoter. Retrovirology.

[13] Liu L, Zhou XM, Yang FF, Miao Y, Yin Y, Hu XJ (2017). TRIM22 confers poor prognosis and promotes epithelial-mesenchymal transition through regulation of AKT/GSK3beta/beta-catenin signaling in non-small cell lung cancer. Oncotarget.

[14] Li L, Qi Y, Ma X, Xiong G, Wang L, Bao C (2018). TRIM22 knockdown suppresses chronic myeloid leukemia via inhibiting PI3K/Akt/mTOR signaling pathway. Cell Biol Int.

[15] Liu M, Kuo F, Capistrano KJ, Kang D, Nixon BG, Shi W (2020). TGF-beta suppresses type 2 immunity to cancer. Nature.

[16] Taniguchi S, Elhance A, Van Duzer A, Kumar S, Leitenberger JJ, Oshimori N (2020). Tumor-initiating cells establish an IL-33-TGF-beta niche signaling loop to promote cancer progression. Science.

[17] Gabitova-Cornell L, Surumbayeva A, Peri S, Franco-Barraza J, Restifo D, Weitz N (2020). Cholesterol pathway inhibition induces tgf-beta signaling to promote basal differentiation in pancreatic cancer. Cancer Cell.

[18] Trinh BQ, Barengo N, Naora H (2011). Homeodomain protein DLX4 counteracts key transcriptional control mechanisms of the TGF-beta cytostatic program and blocks the antiproliferative effect of TGF-beta. Oncogene.

[19] Deane CAS, Brown IR (2018). Knockdown of heat shock proteins HSPA6 (Hsp70B’) and HSPA1A (Hsp70-1) sensitizes differentiated human neuronal cells to cellular stress. Neurochem Res.

[20] Schlesinger MJ (1990). Heat shock proteins. J Biol Chem.

[21] Noonan EJ, Place RF, Giardina C, Hightower LE (2007). Hsp70B’ regulation and function. Cell Stress Chaperones.

[22] Shin SS, Song JH, Hwang B, Noh DH, Park SL, Kim WT (2017). HSPA6 augments garlic extract-induced inhibition of proliferation, migration, and invasion of bladder cancer EJ cells; Implication for cell cycle dysregulation, signaling pathway alteration, and transcription factor-associated MMP-9 regulation. PLoS ONE.

[23] Javelaud D, Alexaki VI, Dennler S, Mohammad KS, Guise TA, Mauviel A (2011). TGF-beta/SMAD/GLI2 signaling axis in cancer progression and metastasis. Cancer Res.

[24] Azar R, Alard A, Susini C, Bousquet C, Pyronnet S (2009). 4E-BP1 is a target of Smad4 essential for TGFbeta-mediated inhibition of cell proliferation. EMBO J.

[25] Chen CR, Kang Y, Siegel PM, Massague J (2002). E2F4/5 and p107 as Smad cofactors linking the TGFbeta receptor to c-myc repression. Cell.

[26] Ikushima H, Miyazono K (2010). TGFbeta signalling: a complex web in cancer progression. Nat Rev Cancer.

[27] Jiang Z, Cao Q, Dai G, Wang J, Liu C, Lv L (2019). Celastrol inhibits colorectal cancer through TGF-beta1/Smad signaling. Onco Targets Ther.

[28] Zhang X, Zhang P, Shao M, Zang X, Zhang J, Mao F (2018). SALL4 activates TGF-beta/SMAD signaling pathway to induce EMT and promote gastric cancer metastasis. Cancer Manag Res.

[29] Chen MT, Sun HF, Li LD, Zhao Y, Yang LP, Gao SP (2018). Downregulation of FOXP2 promotes breast cancer migration and invasion through TGFbeta/SMAD signaling pathway. Oncol Lett.

[30] Derynck R, Zhang YE (2003). Smad-dependent and Smad-independent pathways in TGF-beta family signalling. Nature.

[31] Azim HA, Peccatori FA, Brohée S, Branstetter D, Loi S, Viale G (2015). RANK-ligand (RANKL) expression in young breast cancer patients and during pregnancy. Breast Cancer Res.

